# Sub-perfluoro-n-octane injection of ocular viscoelastic device assisted inverted internal limiting membrane flap for macular hole retinal detachment surgery: a novel technique

**DOI:** 10.1186/s12886-020-01393-1

**Published:** 2020-03-21

**Authors:** Chi-Chun Lai, An-Lun Wu, Hung-Da Chou, Wee Min Teh, Kuan-Jen Chen, Yen-Po Chen, Laura Liu, Yih-Shiou Hwang, Wei-Chi Wu

**Affiliations:** 1 Department of Ophthalmology, Chang Gung Memorial Hospital, No. 5, Fu-Hsing Street, Taoyuan, 333 Taiwan; 2 College of Medicine, Chang Gung University, Taoyuan, Taiwan; 3 Department of Ophthalmology, Mackay Memorial Hospital, Hsinchu, Taiwan; 4 Department of Ophthalmology, Hospital Selayang, Batu Caves, Selangor Malaysia

**Keywords:** Macular hole retinal detachment, Internal limiting membrane, Perfluoro-n-octane, Ocular viscoelastic device

## Abstract

**Background:**

To evaluate the feasibility of a surgical technique using a sub-perfluoro-n-octane (PFO) injection of ocular viscoelastic device (OVD) to stabilize inverted internal limiting membrane (ILM) flap for the treatment of macular hole retinal detachment (MHRD).

**Methods:**

This study was a retrospective, consecutive, interventional case series. Patients who underwent MHRD surgery with sub-PFO injection of OVD to stabilize inverted ILM flap onto the macular hole (MH) were reviewed. The color fundus and optical coherence tomography (OCT) images were collected and evaluated. The best-corrected visual acuity (BCVA) before and after surgery were compared as the functional outcome.

**Results:**

The study included 8 eyes of 8 consecutive patients (mean age: 61.8 ± 7.1 years; mean follow-up period: 9.0 ± 2.5 months). All eyes (100%) achieved successful MH closure; 7 eyes (87.5%) demonstrated complete retinal reattachment, and 1 eye (12.5%) had minimal residual subretinal fluid parafoveally. Of the 8 patients, 7 patients (87.5%) had achieved improvement in BCVA after the primary surgery, whereas 1 eye remained stable. The average BCVA before and after the surgery at the last visit improved from 20/843 (1.63 ± 0.48 logMAR) to 20/200 (1.00 ± 0.39 logMAR) (*P* = 0.016). Anatomically, near-normal foveal contour was noted in five (62.5%) eyes at the final follow-up.

**Conclusions:**

The use of sub-PFO injection of OVD in MHRD surgery could stabilize inverted ILM flaps, achieve good anatomical results and improve postoperative BCVA.

## Background

Macular hole retinal detachment (MHRD) in highly myopic eyes is a vision-threatening complication and could result in significant reduction in visual acuity (VA). MHRD surgical success has been challenging in terms of not only retinal attachment but also MH closure [1]. The ideal modality for treating MHRD remains unknown. Recently, MHRD surgical intervention by pars plana vitrectomy (PPV) with an inverted internal limiting membrane (ILM) flap [2] and its modified techniques have been developed to improve retinal attachment and macular hole (MH) closure rate [3–6].

During the intraoperative ILM manipulation, ocular viscoelastic device (OVD) has been noted to be a safe and effective retinal glue to stabilize ILM flaps [7]. However, application of the OVD directly on the ILM flap in an appropriate volume is a technical challenge. During the application, the OVD may disperse and float. In addition, uniform injection of the OVD on the surface of the ILM flap to ensure that it is flatly attached to the retina is difficult. A novel technique to position inverted ILM flap and ensure its stabilization to the desirable position is strongly needed.

Herein, we develop a novel surgical technique involving the use of sub-perfluoro-n-octane (PFO) injection of OVD to stabilize ILM flap for MHRD treatment. The ILM was peeled and inverted onto the MH under PFO tamponade and then OVD was introduced onto the PFO-retina interface above the inverted ILM flap overlying the MH. This study evaluates the feasibility of this technique for MHRD treatment.

## Methods

This study adhered to the guidelines of the Declaration of Helsinki and was approved by the institutional review board of Chang Gung Memorial Hospital, Taoyuan, Taiwan. Patients with MHRD who underwent PPV with sub-PFO inverted ILM flap plus OVD injection technique with a follow-up duration of over 6 months were retrospectively reviewed in this study. All patients received comprehensive ophthalmologic examinations before and after the surgery including measurement of best-corrected VA (BCVA), color fundus, and optical coherence tomography (OCT) imaging. MH size was measured with the built-in software provided by the manufacturer. BCVA measured using a Snellen chart was converted to the logarithm of minimum angle of resolution (logMAR) for analysis. The classifications of MHRD were based on the extent of retinal detachment (RD) [8]. The Wilcoxon signed-rank test was used for the evaluation of preoperative VA compared with postoperative VA. The data were analyzed by statistical software (SPSS software version 23.0; SPSS, Inc., Chicago, IL, USA) with *P <* .05 considered significant.

### Surgical technique

**Fig. 1:**
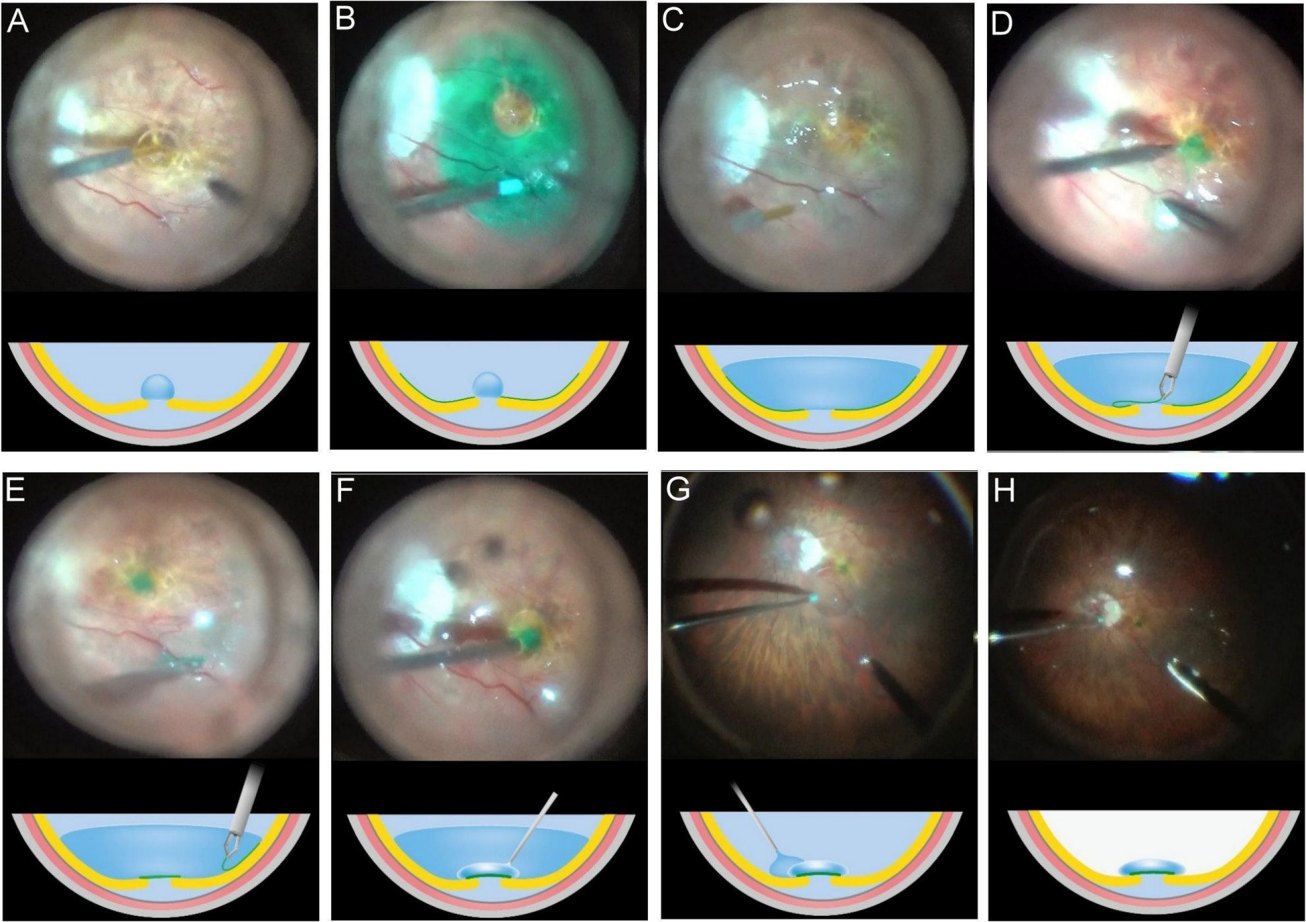
Sub-perfluoro-n-octane (PFO) injection of ocular viscoelastic device (OVD) technique in treating macular hole retinal detachment (MHRD). Cross-sectional schematic diagram (bottom) showing surgical steps corresponding to each photograph (top). **a**, **b** A small PFO bubble was introduced into the eye to protect the fovea, followed by an injection of indocyanine green (ICG) dye. **c** Excess ICG dye was removed and additional PFO was injected to fill the posterior pole. **d** The ILM peel was initiated by forceps under PFO tamponade about 4 disc diameters in size circumferentially, and multiple ILM layers were inverted to cover the MH. **e** Remaining ILM within the macula was intended to peel close to the vascular arcades. **f** A small amount of OVD was then injected under PFO to cover the ILM flap. **g** PFO was completely removed in the fluid phase. **h** Fluid–air exchange was performed, followed by injection of 15% C_3_F_8_


All eyes underwent a 23-gauge PPV (Constellation; Alcon Laboratories, Inc., Fort Worth, TX, USA) with retrobulbar anesthesia. The surgical procedures were performed by a single surgeon (C.-C.L.) and the key steps of this technique are outlined in Fig. 1 (see the video in Additional file 1). After core vitrectomy, pre-macular vitreous cortex was removed using triamcinolone staining and a diamond-dusted membrane scraper (Synergetics, Inc., O’Fallon, MO, USA), as described previously [4]. Approximately 0.1–0.2 ml of PFO (Perfluoron; Alcon Laboratories, Inc., Fort Worth, TX) was introduced as a single bubble above the disc and then the eye was carefully rotated to displace the PFO bubble to cover the MH (Fig. 1a), followed by an injection of indocyanine green (ICG; Daiichi, Tokyo, Japan) in the concentration of 1 mg/ml in 5% glucose water over the macular area to stain the ILM (Fig. 1b). Excess ICG was aspirated and PFO was further injected to cover the macular area slightly beyond the arcade vessels (Fig. 1c). The fluid pressure was lowered to 10 mmHg. The ILM peel was initiated by forceps under PFO tamponade about 4 disc diameters in size circumferentially, and multiple ILM layers were inverted to cover the MH by 23-gauge disposable forceps (Alcon Laboratories, Fort Worth, TX, USA; Fig. 1d). No action of insertion was performed during this maneuver, and no subretinal fluid was drained intentionally. Subsequently, remaining ILM within the macula was intended to peel close to the vascular arcades (Fig. 1e). After the MH defect was covered by inverted ILM flaps, a small drop of 0.05–0.1 mL of OVD (Viscoat; Alcon Laboratories) was subsequently injected gently onto the retina–PFO interface above the ILM flap on the MH area, as shown in Fig. 1f. The peripheral retina was examined circumferentially after peripheral vitrectomy and laser photocoagulation was applied if retinal tears or lattice were present. The PFO was then completely removed in the fluid phase before fluid–air exchange (FAE) was performed (Fig. 1g, h). At the end of surgery, air was replaced with 15% C_3_F_8_ gas. Patients were asked to remain face down for 1 day after surgery and avoid supine position afterward during the follow-up period until the gas was absorbed.

**Additional file 1.** Surgical video that illustrates key steps of sub-perfluoro-n-octane injection of ocular viscoelastic device technique for managing macular hole retinal detachment.


## Results

**Fig. 2:**
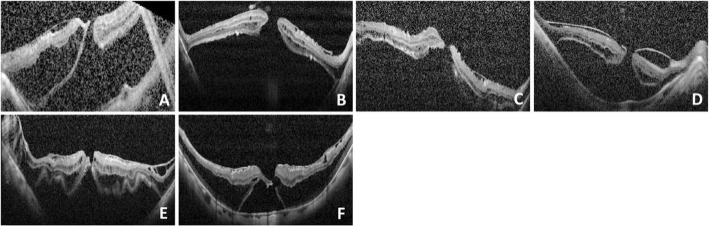
Preoperative OCT images in 6 of the 8 cases. **a**–**f** correspond to patients 1–6. We were unable to take the OCT scans for patient 7 and 8

This study recruited 8 eyes of 8 highly myopic patients with MHRD. Fundus examination indicated that 4 eyes were classified as having type 1 MHRD and that the remaining 4 had type 2 MHRD. The average axial length was 29.87 ± 2.03 mm. The patient population consisted of 5 women and 3 men (mean age: 61.8 ± 7.1 years; mean follow-up period: 9.0 ± 2.5 months). The baseline clinical characteristics of patients are shown in Table 1. The preoperative OCT images are shown in Fig. 2.

**Table 1:** Demographics, Main Clinical Features at Baseline, and Surgical Outcomes for Macular Hole Retinal Detachment

Patient No.	Age (Years)	Sex	Eye	Subtype of MHRD^a^	Axial length (mm)	MH size (μm)	Retinal attachment	MH closed	Final Lens Status	Preoperative BCVA	Final BCVA	Follow-up (month)
1	60–70	F	OS	Type 2	33.10	48	Yes	Yes	Cataract	20/400	20/400	6
2	50–60	F	OD	Type 1	27.56	214	Yes	Yes	Cataract	20/2000	20/400	7
3	50–60	F	OS	Type 2	29.05	105	Yes	Yes	Pseudophakic	20/1000	20/200	10
4	60–70	F	OS	Type 1	27.52	153	Yes	Yes	Pseudophakic	20/2000	20/400	11
5	70–80	M	OS	Type 1	30.50	28	Yes	Yes	Pseudophakic	20/400	20/200	12
6	60–70	M	OS	Type 1	28.58	114	Yes	Yes	Pseudophakic	20/100	20/50	12
7	50–60	M	OD	Type 2	30.81	NA^b^	Yes	Yes	Pseudophakic	20/2000	20/50	7
8	50–60	F	OS	Type 2	31.86	NA^b^	Yes	Yes	Cataract	20/2000	20/400	7

*BCVA* Best-corrected visual acuity, *MH* Macular hole, *NA* Not applicable, *RD* Retinal detachment

^a^ Type 1 indicated RD confined to the macula (within the boundaries of the superior and inferior temporal arcade vessels), and type 2 indicated RD beyond the macula

^b^ No clear OCT images could be obtained preoperatively for measurements

**Fig. 3:**
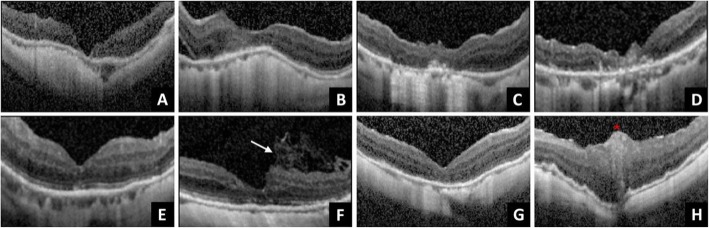
OCT images in the last visit of the 8 cases was shown. **a**–**h** correspond to patients 1–8, and 5 eyes had near-normal foveal contour **(b**, **c**, **e**, **f**, and **g)**. **a** Patient 1 had a steep foveal contour. **b** In patient 2, minimal residual subretinal fluid was noted. **d** Irregular foveal contour was noted in patient 4. **f** In patient 6, multiple-layer ILM flap was not directly on the top of fovea but located at the parafoveal area (arrow). **h** In patient 8, ILM plug appeared to reside within the fovea; the star indicating the ILM plug

MH closure was seen in all 8 eyes (100%) after the initial surgery (Fig. 3). Complete retinal reattachment was achieved in 7 eyes (87.5%), whereas in one eye (12.5%) (Fig. 3b), minimal subretinal fluid at the parafoveal region was noted. During the procedure while performing sub-PFO injection of OVD, we did not observe any complications of entrapped PFO in OVD or subretinal migration of PFO. Postoperative abnormal intraocular pressure episodes did not occur in any eye. Regarding the postoperative restoration of the foveal contour observed through OCT, 5 eyes (62.5%) had near-normal foveal contour (Fig. 3b, c, e, f, and g). Meanwhile, one eye (12.5%) each had MH closure with steep foveal contour (Fig. 3a), irregular foveal contour (Fig. 3d), and ILM flap appeared sunken into the MH (Fig. 3h), respectively. Of the five eyes with near-normal foveal contour, one of them had the ILM flap that was marginally away from the fovea (Fig. 3f). The BCVA was improved in 7 eyes (87.5%), whereas one (12.5%) remained stable and unchanged at the final follow-up examination.

The mean preoperative and postoperative last-visit BCVA improved from 20/843 (1.63 ± 0.48 logMAR) to 20/200 (1.00 ± 0.39 logMAR) (*P* = 0.016). At the end of the follow-up period, apart from the 5 preexisting pseudophakic eyes, the other 3 phakic eyes showed some degree of cataract progression. Residual subretinal fluid (SRF) was noted in one (12.5%) eye even after 7 months follow-up.

## Discussion

Since the inverted ILM flap technique was introduced by Michalewska et al. [2] in 2010, it has been modified and used widely to treat different refractory MH, including MHRD [3]. However, peeling the ILM from the surface of the detached retina and then inverting or covering this fragile ILM flap into the MH can be challenging. In addition, preventing the floating and dislocation of the ILM flap in these eyes is difficult. In this study, our technique may provide some advantages: First, ILM is easier to peel and invert to cover the MH under PFO, which may reduce surgical damage to the fovea. Second, sub-PFO injection of OVD could provide a more precise way to cover and stabilize the ILM flap. Therefore, the combination of PFO-assisted inverted ILM flap and sub-PFO injection of OVD could be an easy, safe, and effective method for MHRD treatment.

Use of PFO to assist the ILM peeling for MHRD was introduced in 2003 by Brazitikos et al. [9]. The researchers demonstrated the stabilizing effect of PFO on the retina after SRF drainage, which makes the stained ILM easier to peel for MHRD treatment. In our surgical technique, we did not intentionally drain the SRF during surgery in all our patients for a few notable reasons. Firstly, some amount of fluid may remain in the subretinal space despite aggressive drainage. Secondly, the shearing forces from aspirating fluid via the macular hole may enlarge the hole and cause further damage to the foveal tissue. Thirdly, OVD is highly viscous and has dispersive property, which may cause it to adhere strongly onto the retinal surface. Thus, the complex of inverted ILM flap and OVD could act as a plug to “seal” the macular hole to obstruct the prolapse of SRF. Lastly, SRF could be reabsorbed eventually [4]. The presence of SRF did not affect the stabilization of retina during ILM peeling in our study. A small amount of SRF may in fact be beneficial to act as a cushion and prevent mechanical damage from the forceps during peeling and inverting of ILM flap onto the MH. Therefore, it is not necessary to drain the SRF for the treatment of MHRD with the assistance of PFO. In addition, a single small PFO bubble is injected at the beginning of surgery before ILM staining. This could prevent the subretinal migration of ICG and reduce the risk of retinal toxicity when we use ICG to stain the ILM.

Inverted ILM flap in MHRD is technically difficult to perform. By using PFO, it is easier to relieve the fragile ILM flap from forceps, and reposition the flap into the MH and prevent flap dislodgement during FAE [10]. In our study, PFO was used to assist the positioning of the ILM flap onto the MH. With PFO, this can be performed in a more controlled manner to prevent the tearing of the ILM flap from the margins of the MH in highly myopic eyes with decreased contrast due to light pigmentation of the fundus. Furthermore, the action of insertion is unnecessary because the inverted ILM could be precisely fixed onto the retinal surface by the high interfacial tension, further reducing the risk of injury to the fovea. The PFO also helped to easily release this fragile ILM flap and improve the speed of surgery.

After the ILM flaps are inverted or transplanted into the MH, the next challenge is to prevent it from floating or dislocating. The original article reported by Michalewska et al. [2] mentioned that approximately 14% of inverted ILM flaps were dislocated during FAE. Even after FAE, the remnant vitreous fluid may soon track down from the periphery toward the MH area. This fluid, combined with the movement of the globe, could produce turbulence, which may dislocate the ILM flap from the MH after surgery. Thus, retinal glue such as OVD to stabilize the ILM flap has been used to resolve the problem [5, 7, 11, 12]. However, OVD application onto MH or ILM is challenging in a fluid-filled eye. The OVD may disperse before coming into contact with the ILM or MH, making it difficult for it to be delivered to the exact area that needs to be covered. Further, despite successful application the ILM flap may be pushed out of the MH by the OVD. The effectiveness of sub-PFO injection of OVD depends on specific gravity of PFO, low viscosity property of PFO, and high interfacial tension between OVD and PFO [13]. The direction of OVD injection is just above the inverted ILM flap to cover it under PFO tamponade. A hydrostatic force due to PFO gravity pushes the retina downwards, which displaces the SRF anteriorly, reducing the risks of injection of OVD into the sub-ILM flap space. Injecting OVD in the presence of PFO is a more controlled way to apply OVD to stabilize the ILM flap and only a very small amount of OVD is needed to cover the MH area and stabilize the ILM flap.

Not only the anatomical closure, but functional outcome in terms of BCVA improvement has become the surgical goal for the treatment of MHRD [14]. And the postoperative restoration of the foveolar architecture was found to be associated with the visual function recovery [6, 15]. The results from previous studies have shown that there were extra material/tissue or irregular ILM contractions within the hole after vitrectomy for MHRD treatment [3, 4, 16]. In our study, we used multiple layers of inverted ILM flap without insertion to provide more scaffolds for MH closure and minimize gliosis. We hypothesize that multilayered ILM act like scaffolds for tissue proliferation, manufacturing a microenvironment that supports correct photoreceptor positioning in direct proximity to the fovea to assume better foveal architectural restoration and consequently better functional restoration. In theory, by using the sub-PFO injection of the OVD technique, OVD could be more precisely positioned above the ILM flap surface by the high interfacial tension and may further reduce the interference of original glial proliferation process. However, the glial proliferation on the fovea remained visible in one of our cases, while others had better foveal restoration.

The current study has several limitations. It was retrospective in design, a small sample size as well as a relatively short follow-up period. And the study did not have a control arm of inverted ILM flap and PFO without OVD. Additionally, using PFO could also increase the cost of the surgery.

## Conclusions

The combination of PFO-assisted inverted ILM flap and sub-PFO injection of OVD to stabilize ILM flap for MHRD treatment is feasible. It facilitates a novel approach to peel ILM on a detached retina, positions the inverted ILM flap to cover the MH, and possibly reduces the surgical damage on the macula. In addition, it also provides a more controlled way to apply OVD onto the ILM flap to stabilize it during surgery. Long-term follow-up of these patients and additional studies assessing the outcome of this technique in a larger cohort are warranted.

## Data Availability

The datasets used and/or analyzed during the current study available from the corresponding author on reasonable request. (E-mail: chichun.lai@gmail.com).

## Abbreviations

BCVA: Best-corrected visual acuity
FAE: Fluid–air exchange
ILM: Internal limiting membrane
ICG: Indocyanine green
logMAR: Logarithm of minimum angle of resolution
MH: Macular hole
MHRD: Macular hole retinal detachment
OVD: Ocular viscoelastic device
OCT: Optical coherence tomography
PPV: Pars plana vitrectomy
PFO: Perfluoro-n-octane
RD: Retinal detachment
SRF: Subretinal fluid
